# Mechanosensitive Piezo1 and Piezo2 ion channels in craniofacial development and dentistry: Recent advances and prospects

**DOI:** 10.3389/fphys.2022.1039714

**Published:** 2022-10-21

**Authors:** Yifan Lin, Jianhan Ren, Colman McGrath

**Affiliations:** Faculty of Dentistry, The University of Hong Kong, Pokfulam, Hong Kong SAR, China

**Keywords:** Piezo1, Piezo2, mechanosensitive ion channels, craniofacial development, dentistry

## Abstract

Mechanical forces play important roles in many biological processes and there is increasing interest and understanding of these roles. Mechanotransduction is the process by which mechanical stimuli are converted to biochemical signals through specific mechanisms, and this results in the activation of downstream signaling pathways with specific effects on cell behaviors. This review systematically summarizes the current understanding of the mechanosensitive Piezo1 and Piezo2 ion channels in craniofacial bone, tooth, and periodontal tissue, presenting the latest relevant evidence with implications for potential treatments and managements of dental and orofacial diseases and deformities. The mechanosensitive ion channels Piezo1 and Piezo2 are widely expressed in various cells and tissues and have essential functions in mechanosensation and mechanotransduction. These channels play an active role in many physiological and pathological processes, such as growth and development, mechano-stimulated bone homeostasis and the mediation of inflammatory responses. Emerging evidence indicates the expression of Piezo1 and Piezo2 in bone, dental tissues and dental tissue-derived stem cells and suggests that they function in dental sensation transduction, dentin mineralization and periodontal bone remodeling and modulate orthodontic tooth movement.

## Introduction

Mechanical stimuli are known to play crucial roles in regulating diverse cellular functions such as cell growth, proliferation, differentiation, adhesion and migration, that affect tissue and organ maintenance (Du et al., 2022). Mechanical cues originate from extracellular and intracellular components, including tensional or compressional forces directly exerted on cells, the stiffness of substrates and the forces generated by cell deformation during differentiation or migration (Kobayashi and Sokabe, 2010; Ning et al., 2021). Mechanosensitive ion channels, which show specificity for Ca^2+^ influx, enable Ca^2+^-mediated signaling under conditions of mechanical stress and convert mechanical signals to electrical signals. The Ca^2+^ currents induced by mechanical stimuli begin within microseconds and end within milliseconds in the continuous presence of stimuli (Murthy et al., 2017). Several major mechanosensitive ion channels have been discovered, including the two-pore-domain potassium channels and members of the Piezo channel family, transient receptor potential superfamily, transmembrane protein 16 superfamily and epithelial sodium channel/degenerin superfamily (Jin et al., 2020). In this review, we systematically review the recent advances in research on Piezo channels in the fields of craniofacial development and dentistry, and outline the potential directions and challenges for future research.

Piezo1 and Piezo2 were identified in 2010 in a neuroblastoma cell line subjected to a strong mechanically induced current (Coste et al., 2010). Using RNA interference, *Fam38A* was found to be required for mechanotransduction; drawing inspiration from the Greek “πίεση” (pίesi, “pressure”), it was renamed Piezo1 (Coste et al., 2010). Abundant Piezo1 expression was identified in the bladder, colon, kidney, lung and skin but relatively low in the dorsal root ganglia (DRG). In contrast, Piezo2 (*Fam38B*), was strongly expressed in the DRG, and other sensory neurons, such as enterochromaffin cells of the gut, endothelial cells in the brain, Merkel cells, and outer hair cell, suggesting a role in somatosensory mechanotransduction (Coste et al., 2010; Anderson et al., 2017). Later studies confirmed that Piezo2 deficiency leads to a marked decrease in proprioception (Woo et al., 2015; Chesler et al., 2016).

**FIGURE 1:**
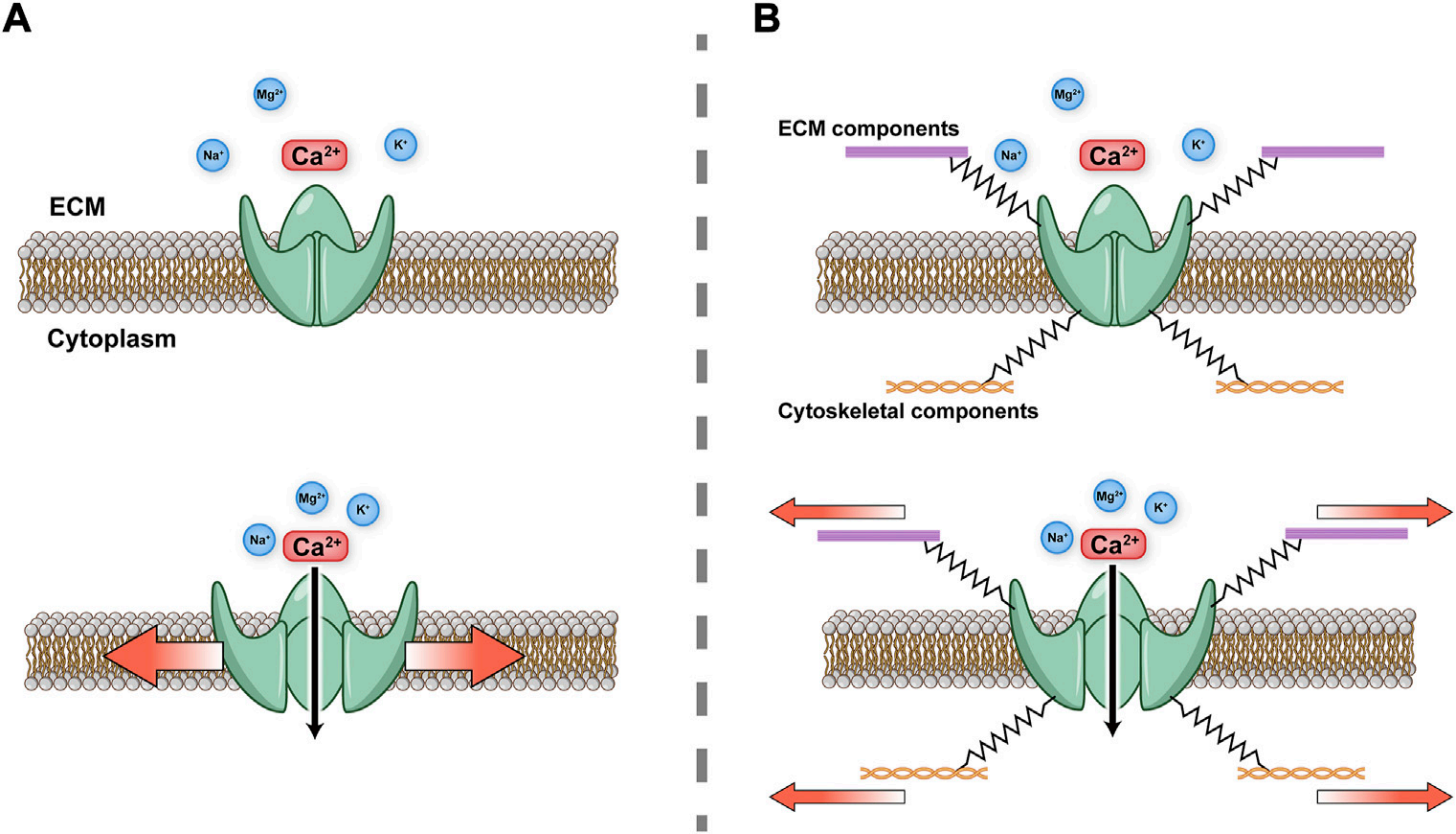
Schematic diagram of Piezo gating modes **(A)** *Force-from-lipids.* Mechanical force activates Piezo channel through the lipid bilayer. **(B)** *Force-from-filaments.* Force acts on cytoskeleton and extracellular matrix (ECM) tethering to the Piezo channel.

Piezo1 and Piezo2 are large transmembrane proteins that share 42% amino acid sequence identity (Coste et al., 2010). High-resolution structural analysis of Piezo1 by cryo-electron microscopy reveals a trimeric three-bladed, propeller-shaped structure with transmembrane domains (Ge et al., 2015). Piezo1 and Piezo2 are large transmembrane proteins that share 42% amino acid sequence identity (Coste et al., 2010). High-resolution structural analysis of Piezo1 by cryo-electron microscopy reveals a trimeric three-bladed, propeller-shaped structure with transmembrane domains (Ge et al., 2015). Piezo1 protein is composed of 38 transmembrane helices in each subunit or “blade”, with 114 transmembrane helices in the whole trimer (Zhao et al., 2018). The central pore modulus is surrounded by transmembrane helix 37 and contains the C-terminal domain (CTD) of each subunit. Piezo2 has a similar structure to Piezo1, but differs from Piezo1 in that there are charged amino acids at the interface between the beam and the CTD. Neutralizing these charged residues leads to an increase in the threshold of the response of Piezo2 to mechanical stress, suggesting a role of the charged residues in facilitating the mechanosensitivity of the Piezo2 channel (Taberner et al., 2019; Wang et al., 2019). The unique transmembrane constriction site in Piezo2 has also been reported to act as a transmembrane gate to restrict ion permeability, whereas the transmembrane gate is dilated in Piezo1 (Wang et al., 2019). Yoda1, the first specific chemical activator of Piezo1 channels identified, activates Piezo1 but not Piezo2, despite their close homology (Syeda et al., 2015). The subsequently reported activators, Jedi1 and Jedi2, activate both human and mouse Piezo1, but fail to activate Piezo2 (Wang et al., 2018). However, both Piezo1 and Piezo2 are inactivated by GsMTx4 (spider venom toxin), which is used to block other mechanosensitive ion channels by altering the membrane curvature surrounding the channel (Murthy et al., 2017). Furthermore, there are two main hypotheses concerning the gating mode of Piezo ion channels. The *force-from-lipids* hypothesis states that tension on the membrane changes the protein conformation by affecting lipid–protein interactions between the membrane and ion channel, which directly activates the channel (Martinac et al., 1990; Kung, 2005; Cox et al., 2016). The *force-from-filaments* hypothesis states that the conformation of a mechanosensitive ion channel changes under mechanical force due to interacting cytoskeletal components or the extracellular matrix (Chalfie, 2009; Katta et al., 2015; Verkest et al., 2022; Wang et al., 2022). The gating mechanisms of Piezo channels are illustrated in Figure 1. Despite a preference for Ca^2+^ influx, other cations such as Mg^2+^, Na^+^ and K^+^ also permeate through Piezo1 (Coste et al., 2010).

Piezo1 and Piezo2 function in various cells and tissues and play active roles in many physiological processes (Li et al., 2014; Pathak et al., 2014; Ranade et al., 2014; Retailleau et al., 2015; Liu et al., 2018; Nonomura et al., 2018). For example, Piezo1 is expressed on embryonic vascular endothelial cells and senses fluid shear stress. The “normal” response to shear stress contributes to endothelial cell alignment, whereas the inactivation of Piezo1 in mice leads to vascular remodeling defects (Ranade et al., 2014). Piezo1 and Piezo2 have also been reported to affect the skeletal system, and Piezo1 deficiency has been shown to impair bone anabolism and induce abnormal responses to external forces (Li et al., 2019; Sun et al., 2019; Wang et al., 2020; Zhou et al., 2020). Furthermore, emerging evidence indicates that Piezo1 and Piezo2 help to regulate dental pain and participate in signaling transduction in dental-derived stem cells, periodontal bone remodeling and orthodontic tooth movement (OTM) (Gao et al., 2017; Sugimoto et al., 2017; Won et al., 2017; Jiang et al., 2021; Cho et al., 2022; Xu et al., 2022).

**FIGURE 2:**
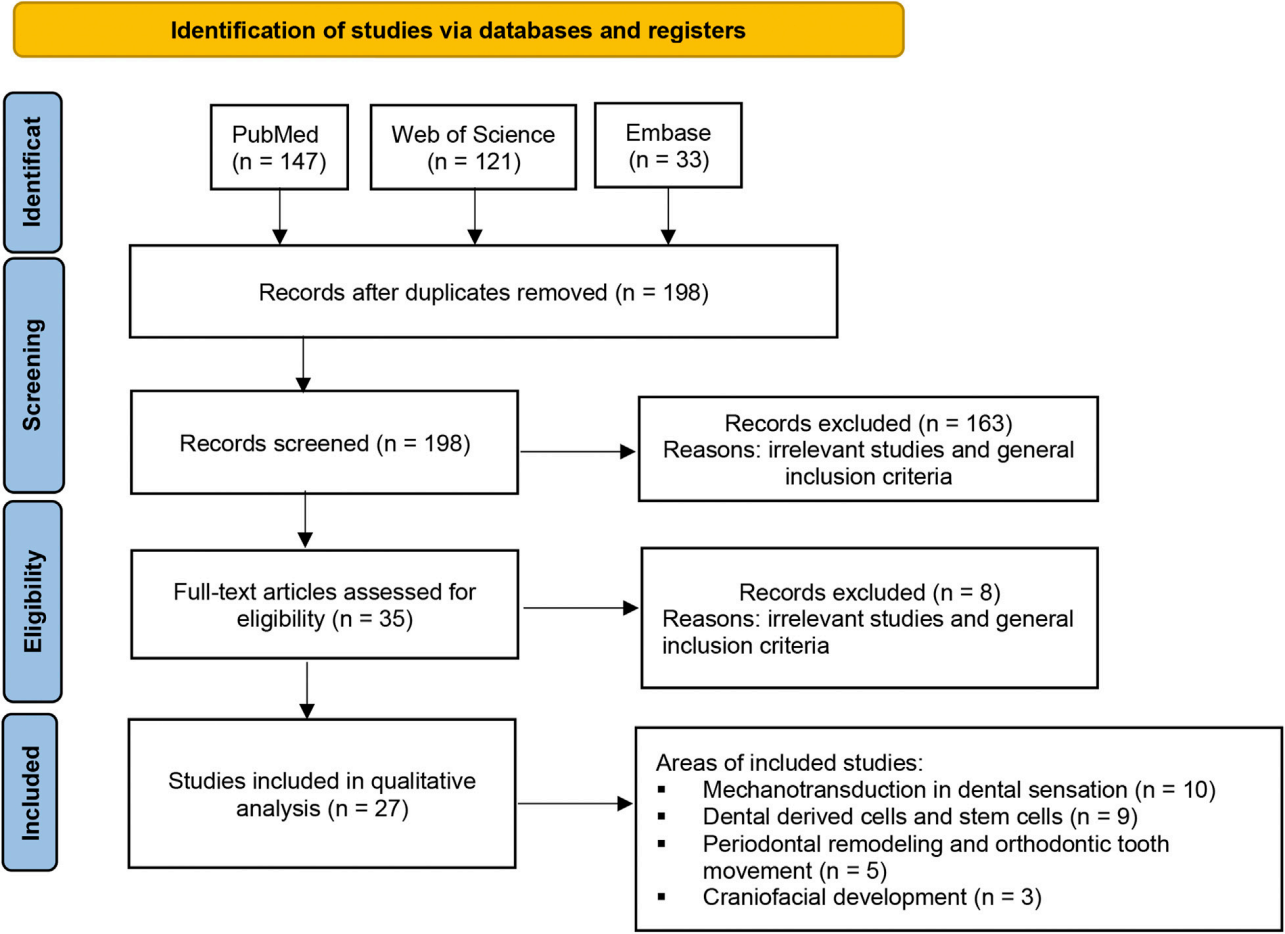
Flow diagram of the study selection process. A total of 301 studies were retrieved based on the search strategy. After removing the duplicates and reviewing the titles and abstracts, 35 records were obtained. After reviewing the full text of these 35 articles, 27 studies met the inclusion criteria and were included in this review.

To systematically review the current evidence on the role of the Piezo1 and Piezo2 ion channels in the fields of craniofacial development and dentistry, a systematic search was conducted in three databases (PubMed, Clarivate Web of Science, and Embase) from the date of their inception to 28 September 2022. The search terms used were as follows: (piezo1 OR piezo2) AND (dental OR dentistry OR tooth OR teeth OR cranial OR craniofacial OR periodontal OR orthodontic OR paediatric OR prosthodontic OR endodontic OR implant OR maxillofacial OR temporomandibular). The inclusion criteria were as follows: 1) studies based on cell, human, or animal models; 2) studies related to the expression or regulation of Piezo1 or Piezo2 in dentistry or craniofacial development; and 3) studies published in English. The exclusion criterion was as follows: reviews, conference abstracts, or editorials. As a result, a total of 301 studies were retrieved. After removing the duplicates and reviewing the titles and abstracts, 35 records were obtained. After reviewing the full text of these 35 articles, 27 studies met the inclusion criteria and were included in this review. A flow diagram of study selection is shown in Figure 2.

## Piezo channels in skeletal and craniofacial development

Mechanical loading of the skeletal system is essential for the development, growth and maintenance of bone. However, the molecular mechanism by which mechanical stimuli are converted into biological signals to regulate bone formation remains largely unclear. Recent advances have demonstrated that Piezo ion channels are greatly involved in bone development and mechanical responses. Because the global deletion of Piezo1 in mice results in embryonic lethality, studies investigating the functions of Piezo1 have employed tissue-specific deletion at various stages of bone development (Zhou et al., 2020). Although Piezo1 and Piezo2 are both expressed in bone tissue, Piezo1 mRNA is detected at higher levels in osteoclasts, osteoblasts and osteocytes (Sugisawa et al., 2020). Murine Piezo1 was found to be strongly expressed in early differentiating osteoblast progenitors, whereas Piezo2 expression was induced during osteoclastogenic differentiation of bone marrow cells (Hendrickx et al., 2021).

### Piezo channels in the growth and development of long bone

The deletion of Piezo1 in osteoblastic lineages in mice results in multiple fractures and severe bone developmental defects (Li et al., 2019; Wang et al., 2020; Zhou et al., 2020; Hendrickx et al., 2021). Conditional deletion of Piezo1 in osteoblasts and osteocytes in mice (*Dmp1-Cre; Piezo1* ^*f/f*^) results in reduced cortical thickness and low bone mineral density compared with control littermates, and these differences increase as the mice mature (Li et al., 2019). Piezo1 controls Wnt1 expression, and thus mediates osteogenesis, *via* the transcriptional co-activators, yes-associated protein (YAP) and PDZ-binding motif (TAZ), which are activated by mechanical signaling. Notably, Zhou et al. (2020) reported that Piezo1-deficient mice (*Prrx1-Cre; Piezo1* ^*f/f*^) exhibited multiple bone fractures in the radius and ulna, suggesting it severely reduces bone formation, whereas Piezo2-deficient mice (*Prrx1-Cre; Piezo2* ^*f/f*^) exhibited grossly normal skeletal development with no bone fractures. Mechanistically, Piezo1/2 mediate the influx of Ca^2+^ by fluid shear stress and extracellular matrix stiffness signals in bone marrow stromal cells. Ca^2+^ influx then stimulates calcineurin, which subsequently promotes the dephosphorylation and complex formation of NFATc1, YAP1, and β-catenin transcription factors. Furthermore, Wang et al. (2020) found that Piezo1-deficient osteoblasts promoted osteoclast differentiation, accounting for the decreased bone mass observed in Piezo1-deficient mice (Wang et al., 2020). Mechanistically, Piezo1 regulates the YAP-dependent expression of collagens, which in turn appears to regulate bone resorption.

Increased osteoclastogenic activity was observed in wild-type but not Piezo1-deficient mice subjected to hind limb unloading to mimic bone loss due to microgravity or disuse, suggesting that Piezo1 in osteoblast-lineage cells regulates bone remodeling by sensing mechanical loading (Wang et al., 2020). This finding may have implications to inform therapeutic strategies for disuse osteoporosis in the settings of prolonged bed rest or long-term exposure to a microgravity environment in space. Similar findings were reported by Zhou et al. (2020). Furthermore, a significant negative correlation of *PIEZO1* and *PIEZO2* expression with age was observed in human bone marrow-derived stem cells from 11 male subjects, suggesting that a reduction in the number of Piezo channels may contribute to bone aging (Zhou et al., 2020). Further elucidation of the cell type- and age-specific functions of Piezo1 and Piezo2 can provide new insights into the underlying cellular and molecular mechanisms by which mechanical forces affect development and health.

### Piezo channels in the growth and development of cranial bone

Notably, the low-bone-mass phenotype of Piezo1-deficient mice appears to be restricted to load-bearing long bones. The results pertaining to less load-bearing cranial bones are controversial, suggesting a differential effect of Piezo1 by bone type. Calvariae from Piezo1-deficient mice are indistinguishable from calvariae from their control littermates at 6 weeks of age, in contrast to substantial differences in the femurs of these mice; this difference may occur because calvariae are less load-bearing than long bones (Wang et al., 2020). Another study by Hendrickx et al. (2021) found that *Runx2* ^*Cre*^ *; Piezo1* ^*f/f*^ mice exhibit no calvarial bone defects at birth nor changes in calvarial thickness or porosity at 12 weeks of age. It was further suggested that *Piezo1* deletion during the early stages of osteoblast differentiation specifically affects bones that are formed through endochondral ossification (Hendrickx et al., 2021). In contrast, Sun et al. (2019) reported that *Piezo1* deficiency results in incomplete closure of cranial sutures in newborn *Ocn* ^*Cre*^ *; Piezo1* ^*f/f*^ mice.

## Piezo channels as mechanical sensors in the dental sensory system

Dental hypersensitivity is “pain derived from exposed dentin in response to chemical, thermal tactile or osmotic stimuli, which cannot be explained as arising from any other dental defect or disease” (Addy and Urquhart, 1992). The hydrodynamic theory is the most widely accepted hypothesis on its pathogenesis, which postulates that mechanical, thermal or chemical changes gives rise to movement of fluid within dentinal tubules, which stimulates the terminals of pulpal nerve fibers, thereby inducing transient acute pain (Brannström, 1963; Brännström, 1992; Dababneh et al., 1999).

Odontoblasts express Piezo1 and Piezo2 channels, and their roles as mechanosensitive transducer cells in the dental sensory system have been widely explored (Khatibi Shahidi et al., 2015; Sato et al., 2018; Miyazaki et al., 2019; Lee et al., 2020; Matsunaga et al., 2021; Cho et al., 2022; Sun et al., 2022). Sun et al. (2022) reported that Piezo1 is expressed in odontoblastic bodies and processes. Piezo1 mediates mechanical transduction in odontoblasts to release adenosine triphosphate (ATP), which transmits external signals to trigeminal ganglion (TG) neurons and generates pain responses. Another electrophysiological study by Sato et al. (2018) found that odontoblasts detect mechanical stimulation *via* Piezo1 and establish neurotransmission with TG neurons. The deformation of odontoblasts by hydrodynamic forces activates Piezo1 channels, which induces the release of ATP and activates P2X3 receptors on myelinated Aδ neurons. P2X3 receptor activation induces an action potential in Aδ neurons to generate dentinal pain. Cho et al. (2022) further suggested that Piezo1-mediated dental mechanotransduction occurs primarily in the axons in the peripheral pulp, and Piezo1 is involved primarily in mediating acute pain elicited by high-threshold mechanical stimuli. Furthermore, a recent study found that Piezo2 is expressed in most pulpal axons, which extensively branch and form an axonal network in the peripheral pulp under the odontoblast layer. These Piezo2^+^ axons in the peripheral pulp and the dentinal tubules may function as low-threshold mechanoreceptors to evoke pain in response to weak mechanical stimuli (Han et al., 2022). These findings suggest that the local application of a selective Piezo1/2 blocker to the peripheral pulp and dentinal tubules may be effective at alleviating dental pain caused by intrapulpal pressure or dentinal fluid movement (Cho et al., 2022; Han et al., 2022).

Mechanical stimulation-induced calcium signaling mediates intercellular signaling networks during odontoblast communication. Matsunaga et al. (2021) suggested dual roles of the Piezo1 ion channel in signal transduction and dentin mineralization in odontoblasts. In their study, the activation of Piezo1 channels by Yoda1 significantly suppressed mineralization, and knockdown of Piezo1 by short hairpin RNA significantly enhanced mineralization. These findings implicate Piezo1 in preventing excessive dentin formation in the pulp chamber in response to mechanical stimuli on the teeth (e.g., mastication).

Piezo2 channels have been identified in sensory DRG neurons and Merkel cells, where they mediate tactile transduction (Wu et al., 2017). Piezo2-expressing neurons display a rapidly inactivating (RI) current in response to mechanical stimuli. Won et al. (2017) identified that Piezo2 occurs mostly in medium to large dental primary afferent (DPA) neurons, where it mediates nociceptive peptidergic transmission when activated. The RI current can be pharmacologically blocked by ruthenium red or small interfering RNA (siRNA)-mediated Piezo2 knockdown. However, smaller and less mechanosensitive RI currents persisted even after siRNA-mediated Piezo2 knockdown, such residual currents remain to be identified in DPA neurons (Won et al., 2017). A summary of the role of Piezo1/2 ion channels in the mechanotransduction of dental sensation is shown in Table 1.

**TABLE 1:** The expression and function of Piezo ion channels in the mechanotransduction of dental sensation.

Study	Ion channel	Expression	Function
Matsunaga et al. (2021)	Piezo1	Odontoblasts	Mediation of mechanotransduction and intercellular odontoblast communication
			Activation of Piezo1 by Yoda1 suppresses dentinogenesis
Cho et al. (2022)	Piezo1	Axons of all types, but primarily in small myelinated axons (Aδ) of the sensory root of the TG	Mediation of acute pain elicited by high-threshold mechanical stimuli
		Unmyelinated axons in the peripheral pulp	
Sun et al. (2022)	Piezo1	Positively expressed in odontoblasts, but weakly expressed in DPSCs	Mediation of mechanotransduction
Khatibi Shahidi et al. (2015)	Piezo2	Ubiquitously expressed in odontoblasts	—
Chung et al. (2016)	Piezo2	Increased expression in TG in rats with masseter inflammation	May be related to mechanical hyperalgesia
Won et al. (2017)	Piezo2	Mostly in medium-to-large-sized DPA neurons	Mediation of mechanotransduction
Krivanek et al. (2020)	Piezo2	A subset of RYR2^+^ cells in the ameloblast layer	—
Lee et al. (2020)	Piezo2	DPA neurons	—
Dhar et al. (2021)	Piezo2	Trigeminal mesencephalic nucleus	Death of neurons due to tooth loss presumably affects masticatory function
Han et al. (2022)	Piezo2	Axons of all types, but primarily in small myelinated (Aδ) axons in the sensory root of the TG	Mediation of mechanosensation and dental pain *via* glutamate signaling
		Small myelinated axons in the radicular pulp and unmyelinated axons in the peripheral pulp	

DPA, dental primary afferent; DPSC, dental pulp stem cells; RYR2, ryanodine receptor type 2; TG, trigeminal ganglion.

## The role of piezo channels in regulating the functions of dental stem cells

Dental-derived mesenchymal stem cells (MSCs) that reside in specialized dental tissues are readily accessible and thus are a promising resource in regenerative medicine due to their self-renewal, multipotency and tissue-specific differentiation potential (Huang et al., 2009). Dental pulp stem cells (DPSCs), the first MSCs isolated from adult human dental pulp, have strong proliferation- and self-renewal abilities (Gronthos et al., 2000). Low-intensity pulsed ultrasound (LIPUS) is considered an effective non-invasive therapeutic tool to enhance hard tissue repair and fracture healing (Harrison et al., 2016). Piezo1 and Piezo2 are expressed in DPSCs, and this expression may be significantly increased by LIPUS treatment (Gao et al., 2017). LIPUS significantly enhances DPSC proliferation, and this effect is inhibited by the Piezo channel blocker ruthenium red *via* the extracellular signal-regulated kinase (ERK) 1/2–mitogen-activated protein kinase (MAPK) pathway (Gao et al., 2017). These findings indicate that Piezo channels help to transduce LIPUS-induced responses in DPSCs and suggest that LIPUS can promote stem-cell-based dental tissue healing. In a more recent study, Mousawi et al. (2020) verified the functional expression of Piezo1 in DPSCs. Specifically, activation of Piezo1 using Yoda1 stimulates cell migration by inducing ATP release and subsequently activating the P2 receptor purinergic and downstream proline-rich tyrosine kinase 2 and MAPK/ERK signaling pathways (Mousawi et al., 2020). These findings reveal novel molecular and signaling mechanisms regulating stem cell migration.

Hydrostatic pressure (HP) generated by interstitial fluid is an important mechanical force in cells. Piezo1 has been reported to detect HP and function as a cell fate determination factor in MSCs by regulating bone morphogenic protein 2 (BMP2) expression (Sugimoto et al., 2017). Specifically, HP activates ERK1/2 and p38 MAPK signaling through Piezo1, thus inducing BMP2 expression and promoting the osteogenic differentiation of MSCs (Sugimoto et al., 2017). MSCs from human exfoliated deciduous teeth (SHEDs), are a readily available source of stem cells, and these are also regulated by HP *via* Piezo1. In SHEDs, Miyazaki et al. (2019) found that HP significantly enhanced osteogenesis and odontogenesis by modulating Wnt/β-catenin signaling. In this process, Piezo1 functions as a mechanotransducer, converting HP signals into intracellular signals during odontoblast differentiation. The activation of Piezo1 by Yoda1 markedly induces WNT16 expression, which further promotes the differentiation, maturation and mineralization of SHEDs (Miyazaki et al., 2019).

The dental follicle, a loose ectomesenchymally derived connective tissue surrounding the developing tooth germ, plays a key role in tooth eruption and contributes extensively to the development of the periodontium (Huang et al., 2009). Human dental follicle cells (DFCs), which are obtained from the dental follicles of unerupted teeth, are multipotent and are considered ideal stem cells for bone tissue engineering (Morsczeck et al., 2005; Yang et al., 2012). A recent study demonstrated the role of Piezo1 in regulating DFCs (Xing et al., 2022). Xing et al. (2022) found that the activation of Piezo1 by Yoda1 increases the proliferation and osteogenic differentiation of human DFCs by mediating Wnt/β-catenin pathway activity. A summary of the role of Piezo1/2 ion channels in the regulation of dental-derived cells and stem cells is shown in Table 2.

**TABLE 2:** The functional roles of Piezo ion channels in dental-derived cells and tissue.

Study	Cell, tissue, or animal model	Ion channel	External stimulation	Outcome	Downstream signaling pathway
Gao et al. (2017)	DPSC	Piezo1 and Piezo2	LIPUS	LIPUS enhanced the proliferation of DPSCs	Piezo-mediated ERK1/2 MAPK signaling
Sugimoto et al. (2017)	SDP11 (human dental pulp-derived stem cell line)	Piezo1	HP	HP promoted the osteogenesis, but inhibited the adipogenesis of SDP11 cells	Piezo1-mediated ERK1/2 and p38 MAPK signaling pathway
Miyazaki et al. (2019)	SHED	Piezo1	HP	HP enhanced the osteogenesis and odontogenesis of SHEDs	Piezo1-mediated Wnt/β-catenin signaling pathway
Mousawi et al. (2020)	DPSC	Piezo1	—	Activation of Piezo1 by Yoda1 stimulated the migration of DPSCs	MEK/ERK signaling pathway
Hasegawa et al. (2021)	OSCC cell line	Piezo1	—	YAP signaling promoted OSCC cell growth *via* Piezo1 activation	ERK1/2 and p38 MAPK signaling pathway
Hu et al. (2022)	PDLSC	Piezo1	LIPUS	LIPUS enhanced the endothelial differentiation and angiogenesis of PDLSCs *via* Piezo1 activation	—
Xing et al. (2022)	DFC	Piezo1	—	Activation of Piezo1 by Yoda1 promoted the osteogenic differentiation of DFCs	Wnt/β-catenin signaling pathway
Zhang et al. (2022)	MCC	Piezo1	Tensile stress	Tension promoted the early chondrogenic differentiation of MCCs *via* IFT88/Piezo1 signaling	—
Wu et al. (2022)	Mandibular condylar cartilage	Piezo1	Inflammation	Increased expression of Piezo1 in the condylar cartilage of rats with temporomandibular joint osteoarthritis	Smad3 signaling pathway
				Inhibition of Piezo1 by GsMTx4 alleviated osteoarthritic inflammation	
Jin et al. (2015)	PDLC	Piezo1	Compressive stress (2.0 g/cm^2^)	Compression increased the osteoclastogenic-inducing ability of PDLCs *via* Piezo1 activation	NF-kB signaling pathway
Zhang et al. (2017)	Murine cementoblast	Piezo1	Compressive stress (2.0 g/cm^2^)	Compression suppressed cementogenesis *via* Piezo1 inhibition	—
Shen et al. (2020)	PDLC	Piezo1	Tensile stress (15% elongation)	Tensile stress transmitted mechanical signals *via* Piezo1 activation	ERK/MAPK pathway
Jiang et al. (2021)	Rat OTM model	Piezo1	40 g orthodontic force	Tensile stress promoted osteogenesis and facilitated OTM *via* Piezo1 activation	—
Xu et al. (2022)	Human PDL tissue, mouse OTM model, and macrophage	Piezo1	Orthodontic force (*in vivo*) and tensile stress (5% elongation, *in vitro*)	LIPUS enhanced the proliferation of DPSCs	Piezo-mediated ERK1/2 MAPK signaling

BMP2, bone morphogenetic protein 2; DPSC, dental pulp stem cell; PDLSC, periodontal ligament stem cell; DFC, dental follicle cell; ERK, extracellular signal-regulated kinase; HP, hydrostatic pressure; IFT, intraflagellar transport protein 88; LIPUS, Low-intensity pulsed ultrasound; MAPK, mitogen-activated protein kinase; MCC, mandibular condyle chondrocyte; MEK, mitogen-activated protein kinase kinase; NF-kB, Nuclear factor kappa B; OSCC, oral squamous cell carcinoma; OTM, orthodontic tooth movement; PDL, periodontal ligament; PDLC, periodontal ligament cell; PDLSC, periodontal ligament stem cell; SHED, stem cells from human exfoliated deciduous teeth.

## Piezo channels in periodontal bone remodeling and orthodontic tooth movement

The periodontal ligament (PDL), a thin layer of fibrous connective tissue between the alveolar bone and cementum, contributes significantly to the development, function and regeneration of tooth-supporting tissues and bone. PDL cells (PDLCs) are mechanosensitive and play a pivotal role in periodontal tissue homeostasis (De Jong et al., 2017; Lin et al., 2021).

While PDLCs function as important mechanotransducers when teeth are subjected to physiological, pathological or external mechanical stress, differential expression of Piezo1 has been observed in PDLCs subjected to tensile or compressive stress (Jin et al., 2015; Shen et al., 2020). Shen et al. (2020) detected significantly increased expression of Piezo1 in PDLCs after 8 h of exposure to tensile stress and suggested that Piezo1 may transmit mechanical signals *via* the ERK signaling pathway. Jin et al. (2015) previously identified that the expression of Piezo1 and osteoclastogenesis-related markers was significantly increased in PDLCs under compressive stress. Inhibition of Piezo1 by GsMTx4 attenuates the ability of PDLCs to induce osteoclastogenesis by suppressing nuclear factor kappa B (NF-kB) signaling pathway activity, indicating that mechanical stress-induced bone resorption is transduced by Piezo1 and mediated by NF-κB signaling (Jin et al., 2015). Notably, PDLCs are known to respond distinctly to different types of mechanical stress—osteogenesis in response to tensile stress and osteoclastogenesis in response to compressive stress (Kanzaki et al., 2002; Jacobs et al., 2013). Nevertheless, the abovementioned studies showed that the expression level of Piezo1 increases, regardless of whether the PDLCs are subjected to tensile or compressive stress (Jin et al., 2015; Shen et al., 2020). Further studies are required to elucidate the role of Piezo1 in PDLCs during mechanical-force-induced bone remodeling.

OTM is a unique mechanical loading-induced process of bone and periodontal remodeling and regeneration (Masella and Meister, 2006). Successful OTM depends on the balance between bone formation and resorption. Using an animal OTM model, Jiang et al. (2021) showed that Piezo1 was activated by orthodontic force on the tension side during OTM and was crucial for inducing the expression of osteogenic-related markers, whereas Piezo1 inhibition by GsMTx4 was shown to inhibit OTM and reduce bone mass. As osteoclast precursors, macrophages regulate osteoclast differentiation and facilitate bone resorption (Teitelbaum, 2000). Bone marrow-derived macrophages express high levels of Piezo1, which can be activated by mechanical stimulation to trigger a proinflammatory response (Solis et al., 2019). Using both human PDL samples and OTM mouse models, a recent study found that mechanical force induces macrophage proliferation *via* the Piezo1–AKT–cyclin D1 axis (Xu et al., 2022). Moreover, inhibition of Piezo1 by GsMTx4 effectively inhibits tensile stress-induced macrophage proliferation and thus suppresses OTM.

External root resorption is the most common sequela of OTM and occurs as a result of cementum remodeling under high compressive stress (Chan and Darendeliler, 2006). By studying murine cementoblasts, Zhang et al. (2017) found that cementogenesis is suppressed by compressive stress and further inhibited by siRNA-mediated Piezo1 knockdown. Furthermore, decreased expression of osteoprotegerin, a cementogenic activity marker, is strongly correlated with Piezo1 mRNA expression, suggesting that Piezo1 plays a role in cementogenesis and may be a therapeutic target to prevent root resorption during OTM (Zhang et al., 2017).

## Conclusion and future perspectives

The mechanosensitive ion channels Piezo1 and Piezo2 are expressed broadly in human tissues and are closely related to several physiological and pathological processes. This article systematically reviews the current evidence for the roles of Piezo1 and Piezo2 channels in craniofacial development and the dental field. These channels are vital for skeletal and craniofacial bone development. Emerging evidence suggests that both Piezo1 and Piezo2 can help to regulate the physiological activities of dental stem cells, the dental sensory system, periodontal bone remodeling and OTM. However, the precise mechanism whereby Piezo1 and Piezo2 regulate these physiological and pathological processes and the associated signaling pathways require further investigation. Moreover, most previous studies have been cell or animal studies. Clinical trials or case reports of Piezo1/2 as a therapeutic intervention strategy are still lacking. Further research is required to investigate the application of a Piezo1/2-targeting strategy. Such research would offer promising solutions to both systemic and dental conditions, and provide insights into periodontal bone remodeling and orthodontic treatment.
